# Prehospital blood pressure lowering in patients with ultra-acute presumed stroke: A systematic review and meta-analysis

**DOI:** 10.1371/journal.pone.0326494

**Published:** 2025-07-16

**Authors:** Yuyang Liu, Yaheng Tan, Jun Wan, Yangchun Xiao, Qiwen Chen, Yuxin Zheng, Chengli Tian, Xinyue Wang, Wenhao Xu, Xueying Yu, Dianxiang Lu

**Affiliations:** 1 Center for Evidence-based Medicine, Clinical Medical College and Affiliated Hospital of Chengdu University, Chengdu, Sichuan, China; 2 Zunyi Medical University, Zunyi, China; 3 Department of Neurosurgery, Clinical Medical College and Affiliated Hospital of Chengdu University, Chengdu, Sichuan, China; 4 Department of Critical Care Medicine, Clinical Medical College and Affiliated Hospital of Chengdu University, Chengdu, Sichuan, China; Birjand University of Medical Sciences, IRAN, ISLAMIC REPUBLIC OF

## Abstract

**Objective:**

High blood pressure frequently occurs in the setting of acute stroke and is associated with worse prognoses. However, it is still uncertain whether initiating blood pressure-lowering therapy in the prehospital phase after stroke onset can enhance outcomes for patients with undifferentiated acute stroke.

**Methods:**

We conducted a search of the PubMed, Embase, and Cochrane databases to identify randomized controlled trials investigating prehospital blood pressure lowering interventions for presumed ultra-acute stroke (within <6 hours). The primary outcome analyzed was the 90-day modified Rankin Scale (mRS), while mortality was considered a secondary outcome.

**Results:**

This meta-analysis included four studies with a total of 3912 patients. The pooled data revealed no significant difference in poor functional outcomes at 90 days (RR = 0.97, 95% CI: 0.92-1.02) or mortality rates (RR = 1.02, 95% CI: 0.90-1.15) between the group receiving blood pressure lowering treatment and the control or placebo group.

**Conclusions:**

In patients with ultra-acute presumed stroke, prehospital blood pressure lowering treatment within 6 hours of stroke did not improve clinical outcomes (PROSPERO: CRD42024557505).

## Introduction

Worldwide, stroke ranks the second leading cause of death and the third leading cause of disability [1]. Thus, a timely and appropriate response to acute stroke is crucial [2].

High blood pressure is commonly seen during the acute phase of ischemic stroke or cerebral hemorrhage, with over 60% of patients showing an increase in blood pressure within the first hour after stroke onset [3,4]. This rise in blood pressure has been associated with poorer functional outcomes and a higher risk of mortality [5,6]. Current guidelines advocate for lowering blood pressure in patients experiencing acute intracerebral hemorrhage [7,8]. Several blood pressure-lowering agents are commonly used in this context, including glyceryl trinitrate and urapidil, both of which act through distinct mechanisms. Glyceryl trinitrate is an organic nitrate that works by releasing nitric oxide (NO), which induces vasodilation of both arteries and veins, thereby reducing systemic vascular resistance and lowering blood pressure. Urapidil, on the other hand, is an alpha-1 adrenergic receptor antagonist and 5-HT1A receptor agonist that reduces peripheral vascular resistance by blocking alpha-1 receptors [7]. However, the optimal management of blood pressure in the prehospital setting remains unclear [9,10]. Prehospital blood pressure management may be a reasonable strategy to improve their prognosis because of limiting hematoma expansion and minimizing ongoing damage to brain tissue [11,12].

Several randomized controlled trials (RCT) [12–16] have investigated the effects of prehospital blood pressure reduction in patients with ultra-acute presumed stroke. All the results indicated that early blood pressure reduction after stroke does not lead to improved functional outcomes or reduced mortality. A prior meta-analysis [23] reinforced this finding, reporting no significant effect on short- or long-term mortality in patients who did not undergo reperfusion therapy. However, that meta-analysis was constrained by imprecision and inconsistency, largely due to the inclusion of a small number of studies with limited sample sizes. Importantly, few investigations have specifically addressed the feasibility and clinical impact of initiating blood pressure management during the ultra-acute prehospital phase — defined as within 6 hours of stroke onset, prior to hospital admission. Given the potential for individual trials to be underpowered, increasing the risk of type II error, our study aimed to address this evidence gap. We conducted a systematic review, meta-analysis, and trial sequential analysis to assess the effects of prehospital blood pressure-lowering interventions on the prognosis of patients suspected of having acute stroke.

## Methods

### Protocol and guidance

This study adhered to the established guidelines for conducting systematic reviews and network meta-analyses [17,18]. Additionally, the research protocol was registered with PROSPERO (CRD42024557505).

### Search strategy

A comprehensive search of the PubMed, Embase, and Cochrane Library databases was conducted from inception to June 2, 2024, to identify relevant studies on early blood pressure lowering therapy and its impact on outcomes in acute stroke patients. Two reviewers (Q.C. and Y.Z.) independently assessed the eligibility of the studies. The search strategy used to obtain studies from these electronic databases employed various keywords and the Boolean operators “AND” and “OR” (Table S1, Supplemental e-materialin in S1 File). References within the identified articles were further examined to find any additional relevant studies.

### Inclusion and exclusion criteria

Studies were considered eligible and included if they met all the following criteria: (1)RCT documenting patients with a diagnosis of presumed acute stroke; (2) the intervention was prehospital blood pressure lowering therapy within six hours, and the control group was treated with placebo or standard contemporaneous guideline blood pressure; (3) studies reported on adverse outcomes (modified Rankin Scale (mRS)>2) and mortality in people with acute stroke after early blood pressure lowering therapy; (4) studies published in English.

The exclusion criteria were as follows: (1) studies that only reported ischemic stroke or hemorrhagic stroke were not included; (2) no functional outcomes or lack of extractable adverse outcomes and mortality; (3) unavailability of the full text, inclusion of meta-analyses, reviews, letters, or duplicate studies; (4) studies focusing on non-human subjects.

### Date extraction

Two researchers (Y.L. and Y.T.) independently screened and retrieved the studies, resolving any disagreements through discussion with a third reviewer. The extracted data included the first author, year of publication, country, study design, sample size, age, gender, mRS, and mortality rates. The primary goal of this systematic review and meta-analysis was to explore the link between early blood pressure lowering therapy and functional outcomes in patients with suspected acute stroke. The authors employed an electronic data extraction form to compile relevant information from each study.

### Confidence of evidence

Two independent reviewers (Y.L. and Y.T.) assessed the risk of bias in the included studies using the Risk of Bias tool, which evaluates five key domains [19]. Each trial was rated for bias risk as low, high, or with some concerns. The certainty of the evidence was evaluated using the Grading of Recommendations, Assessment, Development, and Evaluation (GRADE) framework, following established guidelines [20]. Discrepancies were resolved by consensus or by consulting a third reviewer (X.Y.) when needed.

### Assessment of risk of bias

The methodological quality of the included trials was evaluated by two independent reviewers (Y.L. and Y.T.) using the Cochrane Risk of Bias tool, which assesses bias across seven specific domains [19]. Each trial was given a score reflecting the level of bias risk—low, high, or unclear. Any differences in assessments between the reviewers were resolved through discussion, and when necessary, a third reviewer (X.Y.) provided the final decision.

### Statistical analysis

Statistical analysis was conducted using Review Manager 5.3 software. Data on mRS > 2 and mortality at 90 days were directly extracted from the studies. Heterogeneity was evaluated using the Cochrane *Q*-test and *I*² statistics, which quantify the proportion of variability due to heterogeneity rather than chance, with values over 60% indicating significant heterogeneity. A random-effects model was applied to pool the relative risk across studies. Statistical significance was determined using two-tailed *P*-values, with *P* < 0.05 considered statistically significant. A forest plot was generated to visually display heterogeneity and the overall effect size. Trial sequential analysis was conducted using a 20% reduction or increase in RR to predict the intervention effects for the primary outcome.

### Subgroup analysis

Subgroup analyses were conducted to evaluate the functional outcomes of patients with ultra-early acute stroke following blood pressure reduction therapy. The aim was to explore differences in outcomes across various subgroups. The subgroups were defined based on (1) age (≤70 vs. > 70 years), (2) type of blood pressure-lowering medication (glyceryl trinitrate vs. urapidil), (3) time to treatment initiation (≤3 hours vs. > 3 hours from symptom onset), and (4) baseline systolic blood pressure (SBP < 140 mmHg vs. SBP ≥ 140 mmHg). These subgroup classifications were chosen to assess potential variations in treatment response and identify specific patient populations that might benefit from early blood pressure management.

### Sensitivity analysis

We conducted a sensitivity analysis using the following methods: (1) acute stroke patients; (2) excluding studies with high risk of bias; (3) excluding studies with high-percentage.

## Result

### Study selection and characteristics

**Fig 1:**
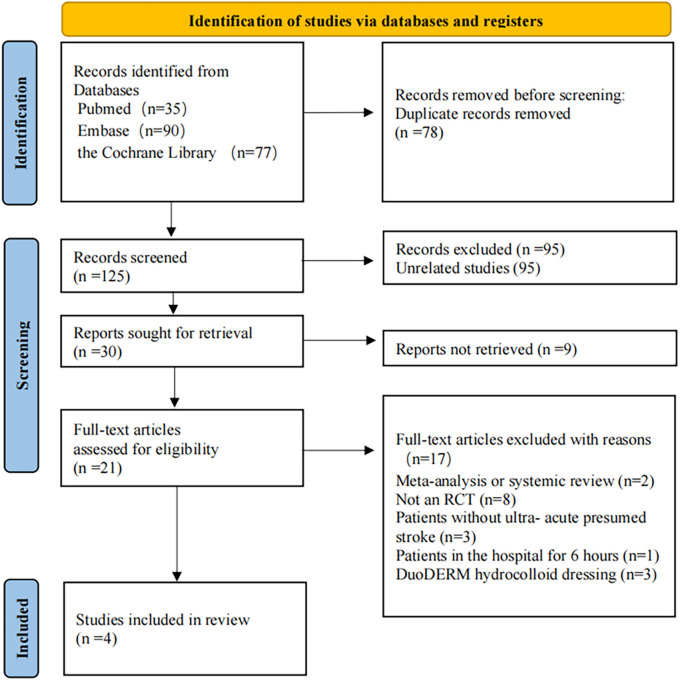
Preferred reporting items for systematic reviews and meta-analyses flow diagram to search and identify included studies.

The initial search identified 202 records. (Table S4, Supplemental e-material in S1 File) After removing duplicates, 125 unique studies remained. These records were carefully evaluated by screening titles, abstracts, and full texts. From this thorough screening process, four trials were included in the final analysis (Fig 1).

Table 1 summarizes the characteristics of the trials, which were published between 2013 and 2024, with sample sizes ranging from 41 to 2,404 participants.

**Table 1:** Characteristics of the studies included in the meta-analysis.

Study	Year	Sample	Mean Age (SD)	Female (%)	Medicine	The time to start treatment at the onset of symptoms	SBP in the included population (mm Hg)
INTERACT4	2024	2404	70.0(12.0)	38.3%	urapidil	within 2 hours	SBP ≥ 150
MR ASAP	2022	325	72.0(13.0)	47.4%	Glyceryl Trinitrate	within 3 hours	SBP > 140
RIGHT-2	2019	1149	72.3(14.6)	48.3%	Glyceryl Trinitrate	within 4 hours	SBP ≥ 120
RIGHT	2013	41	76.9(12.3)	46.3%	Glyceryl Trinitrate	within 4 hours	SBP ≥ 140

SBP: systolic blood pressure.

### Risk-of-bias assessments

Risk-of-bias assessments are presented in Supplemental e-material S1 and S2 Figs in S1 File. One trial [12] was assessed as having a low risk of bias, two trials [13,14] were categorized as having unclear risk, and one trial [15] was considered to have a high risk of bias, primarily due to the use of an open-label design; however, the researchers responsible for analyzing the primary endpoint were blinded.

### Confidence of evidence

The quality of the evidence for primary and secondary outcomes assessed using GRADE was considered to be moderate (Table S2, Supplemental e-material in S1 File).

### The primary outcome: Functional outcome

**Fig 2:**
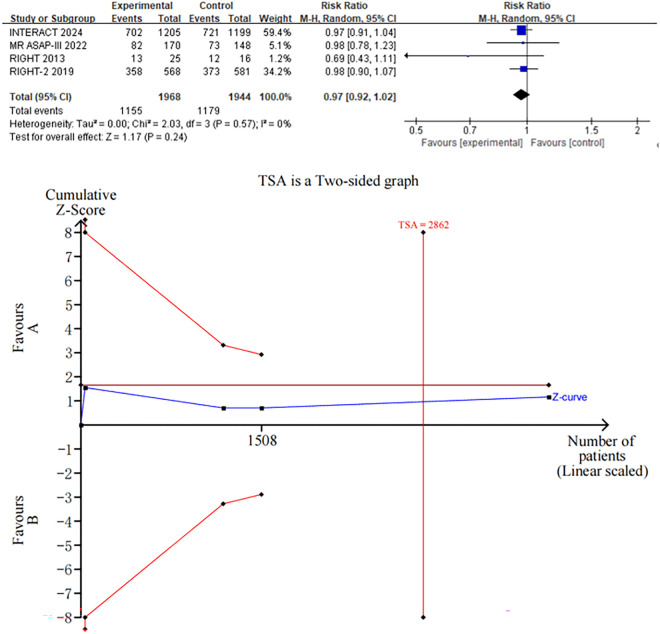
Forest plots of 90-day mRS scores, along with its trial sequential analysis.

Four trials [12–15] reported functional outcomes using the seven-level mRS score 90 days post-randomization. Heterogeneity testing revealed no statistical heterogeneity among these trials (Chi-square = 2.03, *I*² = 0%, *P* = 0.57). Our analysis showed no significant association between early blood pressure reduction and adverse outcomes at 90 days compared to the control/placebo group (RR = 0.97, 95% CI: 0.92–1.02; Fig 2). The trial sequential analysis indicated that 2,862 of the required 3,912 patients had been accrued.

### The secondary outcome: Mortality

**Fig 3:**
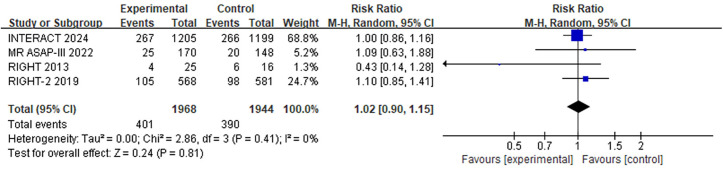
Forest plots of 90-day mortality.

Four trials reported 90-day mortality after blood pressure lowering therapy. In this study, random effect model and RR were employed to combine the findings from 4 RCTs. Our findings suggest that there is no difference in 90-day mortality between the blood pressure lowering treatment group and the control/placebo group (RR = 1.02, 95%CI: 0.90–1.15; Fig 3).

### Subgroup analysis

To detect potential heterogeneity, subgroup analyses were stratified based on the average age, blood pressure lowering medications, time to start treatment, and high blood pressure cut-off value for treatment (Fig S3, Supplemental e-material in S1 File). The results of the subgroup analyses indicated that none of the examined factors significantly contributed to heterogeneity, as all P for interaction values were greater than 0.05. This includes average age (age ≤ 70 vs. age > 70, *P* for interaction = 0.92), blood pressure lowering medications (glyceryl trinitrate vs. urapidil, *P* for interaction = 0.92), time to start treatment (time ≤ 3h vs. time > 3h, *P* for interaction = 0.62), and high blood pressure cut-off value for treatment (Systolic Blood Pressure<140mmHg vs. Systolic Blood Pressure≥140 mmHg, *P* for interaction = 0.69).

### Sensitivity analysis

We excluded high-risk articles and high-proportion articles to examine the effect of a single data set on pooled or undesirable functional outcomes (Table S3, Supplemental e-material in S1 File). The combined RR and its 95% CIs remained largely unaffected, confirming the reliability and robustness of this study’s results.

## Discussion

The findings of this systematic review and meta-analysis indicate that initiating blood pressure–lowering treatment in the prehospital setting within six hours of stroke onset has no significant impact on functional outcomes or mortality rates among patients with ultra-acute presumed stroke.

Most previous meta-analyses [21,22] have indicated that initiating antihypertensive treatment within one week of an acute stroke does not improve functional outcomes in these patients. However, these studies primarily focus on the subacute and early post-stroke phase, while the impact of blood pressure management in the prehospital ultra-acute phase remains largely unexplored [16]. A meta-analysis [23] suggested that high blood pressure management may improve the prognosis of both ischemic and hemorrhagic stroke if initiated within 6 hours of stroke onset. However, the meta-analysis [23] was limited by imprecision and inconsistency, as it considered only two studies (ENOS and RIGHT) [13,16] with a total of 314 patients, which had contradictory findings. Additionally, the larger ENOS trial, conducted in a hospital setting with defined stroke subtypes, may have introduced bias into the overall findings. Given these limitations, high-quality evidence on prehospital blood pressure management in the ultra-acute phase is needed. Our study addresses this gap by analyzing a larger sample with exclusively prehospital interventions, minimizing in-hospital confounding. By systematically assessing the impact of early antihypertensive treatment on clinical outcomes, our findings contribute to a more comprehensive understanding of optimal blood pressure management strategies in the critical early hours following stroke onset.

Our meta-analysis offers several key strengths that enhance the robustness and clinical relevance of our findings. First, by analyzing a larger pooled sample size (3,912 patients), our meta-analysis minimizes the risk of Type II error, enhances statistical power, and reduces the likelihood of failing to detect a true effect. Second, the use of trial sequential analysis (TSA) strengthens the validity of our conclusions by ensuring an adequate information size, thereby reducing the risk of false-negative results. Third, unlike previous reviews that included mixed hospital and prehospital interventions, our study specifically focuses on the ultra-acute prehospital phase (within 6 hours before hospital arrival), providing clearer insights into the feasibility and effectiveness of early blood pressure control strategies. Finally, the clinical implications of our findings are significant. Given that prehospital blood pressure lowering within this critical period did not improve functional outcomes, routine implementation of this approach should be reconsidered. However, our results highlight the need for further investigation into individualized treatment strategies, particularly in subgroups with different stroke types and severity, to refine patient selection criteria and optimize treatment protocols for improved outcomes. There are several limitations to consider when interpreting these findings. First, most of the data are derived from the INTERACT4 trials, which may disproportionately influence the overall results. Second, the antihypertensive medications used across the trials were not uniform, potentially contributing to variability in the functional outcomes. Third, differences in the timing and thresholds for initiating blood pressure–lowering interventions across studies may have introduced significant heterogeneity in the outcomes. Fourth, two of the trials were open-label, and one was single-blind. Although each trial employed an independent blinded assessor to record clinical outcomes, the possibility of observer bias cannot be entirely excluded. Finally, one limitation of the analysis is that it did not account for the speed or magnitude of blood pressure reduction, where potential risks, such as end-organ ischemia, and benefits, such as reduced hematoma expansion, could be closely balanced.

## Conclusion

Our meta-analysis suggests that early blood pressure lowering therapy has no benefit on clinical outcomes in people with ultra-acute presumed stroke.

## Supporting information

S1 File Supplemental e-material.docx. (DOCX)

S2 File The PRISMA 2020 checklist is provided as supporting information, outlining the completeness of our study design, literature screening, and reporting in accordance with PRISMA guidelines. (DOCX)
