# Supplementary material for: Prehospital blood pressure lowering in patients with ultra-acute presumed stroke: A systematic review and meta-analysis
Source: PLoS One. 2025 Jul 16;20(7):e0326494. doi: 10.1371/journal.pone.0326494 (PMC12266422; doi:10.1371/journal.pone.0326494)
Supplement: S1 File — (DOCX) [file pone.0326494.s001.docx]

**Supplemental e-material**

| Table S1: Search strategy  Table S2. Summary of findings and strength of evidence  Table S3. Sensitivity analysis of meta-analysis  Table S4: Excluded Studies |
| --- |
| Figure S1: Risk of bias summary |
| Figure S2: Risk of bias graph  Figure S3: Subgroup analysis of the association between ambulance-intensive blood pressure lowering therapy and poor prognosis |

Table S1: Search strategy.

| **PubMed** | | |
| --- | --- | --- |
|  | "Stroke"[Mesh] | 184951 |
|  | "Blood Pressure"[Mesh] | 314390 |
|  | ((Apoplexy[Title/Abstract]) OR (Cerebral Stroke[Title/Abstract]) OR (Cerebrovascular Accident[Title/Abstract]) OR (Cerebrovascular Apoplexy[Title/Abstract]) OR (Vascular Accident, Brain[Title/Abstract]) OR (CVA (Cerebrovascular Accident)[Title/Abstract]) OR (Cerebrovascular Accident, Acute[Title/Abstract]) OR (Cerebrovascular Stroke[Title/Abstract]) OR (Stroke, Acute[Title/Abstract]) OR (Acute Cerebrovascular Accident[Title/Abstract]) OR (Acute Cerebrovascular Accidents[Title/Abstract]) OR (Acute Stroke[Title/Abstract]) OR (Acute Strokes[Title/Abstract]) OR (Apoplexy, Cerebrovascular[Title/Abstract]) OR (Brain Vascular Accident[Title/Abstract]) OR (Brain Vascular Accidents[Title/Abstract]) OR (CVAs (Cerebrovascular Accident)[Title/Abstract]) OR (Cerebral Strokes[Title/Abstract]) OR (Cerebrovascular Accidents[Title/Abstract]) OR (Cerebrovascular Accidents, Acute[Title/Abstract]) OR (Cerebrovascular Strokes[Title/Abstract]) OR (Stroke, Cerebral[Title/Abstract]) OR (Stroke, Cerebrovascular[Title/Abstract]) OR (Strokes[Title/Abstract]) OR (Strokes, Acute[Title/Abstract]) OR (Strokes, Cerebral[Title/Abstract]) OR (Strokes, Cerebrovascular[Title/Abstract]) OR (Vascular Accidents, Brain[Title/Abstract])) | 478692 |
|  | ((blood pressure[Title/Abstract]) OR (blood tension[Title/Abstract]) OR (Diastolic Pressure[Title/Abstract]) OR (intravascular pressure[Title/Abstract]) OR (normotension[Title/Abstract]) OR (Pressure, Blood[Title/Abstract]) OR (Pressure, Diastolic[Title/Abstract]) OR (Pressure, Pulse[Title/Abstract]) OR (Pressure, Systolic[Title/Abstract]) OR (Pressures, Systolic[Title/Abstract]) OR (Pulse Pressure[Title/Abstract]) OR (Systolic Pressure[Title/Abstract]) OR (vascular pressure[Title/Abstract])) | 389249 |
|  | ambulance[Title/Abstract] | 12870 |
|  | #1 OR #3 | 478692 |
|  | #2 OR #4 | 533466 |
|  | #5 AND #6 AND #7 | 71 |
|  | ((clinical[tiab] AND trial[tiab]) OR "clinical trials as topic"[mesh] OR "clinical trial"[pt] OR random*[tiab] OR "random allocation"[mesh] OR "therapeutic use"[sh]) | 6616315 |
|  | #8 AND #9 | 35 |
| **EMBASE** | | |
| 1. | 'ambulance'/exp | 19315 |
| 2. | 'cerebrovascular accident'/exp | 467606 |
| 3. | 'accident, cerebrovascular':ab OR 'acute cerebrovascular lesion':ab OR 'acute focal cerebral vasculopathy':ab OR 'acute stroke':ab OR 'apoplectic stroke':ab OR 'apoplexia':ab OR 'apoplexy':ab OR 'blood flow disturbance, brain':ab OR 'brain accident':ab OR 'brain attack':ab OR 'brain blood flow disturbance':ab OR 'brain insult':ab OR 'brain insultus':ab OR 'brain vascular accident':ab OR 'cerebral apoplexia':ab OR 'cerebral insult':ab OR 'cerebral stroke':ab OR 'cerebral vascular accident':ab OR 'cerebral vascular insufficiency':ab OR 'cerebro vascular accident':ab OR 'cerebrovascular arrest':ab OR 'cerebrovascular failure':ab OR 'cerebrovascular injury':ab OR 'cerebrovascular insufficiency':ab OR 'cerebrovascular insult':ab OR 'cerebrum vascular accident':ab OR 'cryptogenic stroke':ab OR 'cva':ab OR 'insultus cerebralis':ab OR 'ischaemic seizure':ab OR 'ischemic seizure':ab OR 'stroke':ab OR 'thrombotic stroke':ab OR 'cerebrovascular accident':ab | 505136 |
| 4. | 'ambulances':ab OR 'emergency ambulance':ab OR 'emergency car':ab OR 'emergency vehicle':ab OR 'ambulance':ab | 19075 |
| 5. | 'blood pressure'/exp | 776871 |
| 6. | 'blood pressure':ab OR 'blood tension':ab OR 'diastolic pressure':ab OR 'intravascular pressure':ab OR 'normotension':ab OR 'pressure, blood':ab OR 'pressure, diastolic':ab OR 'pressure, pulse':ab OR 'pressure, systolic':ab OR 'pressures, systolic':ab OR 'pulse pressure':ab OR 'systolic pressure':ab OR 'vascular pressure':ab | 531002 |
| 7. | 'clinical':ti,ab AND 'trial':ti,ab OR 'clinical trial'/exp OR random* OR 'drug therapy':lnk | 7421518 |
| 8. | #1 OR #4 | 26084 |
| 9. | #2 OR #3 | 655592 |
| 10. | #5 OR #6 | 936560 |
| 11. | #7 AND #8 AND #9 AND #10 | 90 |
| **COCHRANE CENTRAL** | | |
| 1. | MeSH descriptor: [Ambulances] in all MeSH products | 258 |
| 2. | MeSH descriptor: [Stroke] explode all trees | 17732 |
| 3. | MeSH descriptor: [Blood Pressure] explode all trees | 34892 |
| 4. | ambulance | 1331 |
| 5. | stroke | 89364 |
| 6. | blood pressure | 139193 |
| 7. | #1 OR #4 | 1411 |
| 8. | #2 OR #5 | 89750 |
| 9. | #3 OR #6 | 139647 |
| 10. | #7 AND #8 AND #9 | 79 |
| 11. | Randomized Controlled Trials as Topic OR randomized controlled trial OR controlled clinical trial OR randomized OR randomly OR trial | 1754803 |
| 12. | #10 AND #11 | 77 |

Table 2. Summary of findings and strength of evidence.

| Outcome | NO. Of patients  (Trials) | Relative Effect | Absolute effect estimates (per 1000) | Quality of the evidence |
| --- | --- | --- | --- | --- |
| The primary outcome | | | | |
| Functional dependency (mRS) at 90 days | 3912(4) | RR=0.97; 95% CI (0.92–1.02) | 603(572 to 634) | Moderate^&^ |
| The secondary safety outcome | | | | |
| Mortality at 90 days | 3912(4) | RR=1.02; 95% CI (0.90–1.15) | 199(175 to 224) | Moderate^&^ |

CI: confidence interval; RR: risk ratio

*Inconsistency

& Risk of bias

Table 3. Sensitivity analysis of meta-analysis.

|  | **NO. Patients(trials)** | **RR** | **95%CI** |
| --- | --- | --- | --- |
| **All trials** | 3912(4) | 0.97 | 0.92,1.02 |
| **Acute stroke patients** | 3411(4) | 0.95 | 0.87,1.05 |
| **Excluding a study with high risk** | 3594(3) | 0.97 | 0.92,1.02 |
| **Excluding a study with high percentage** | 1508(3) | 0.97 | 0.89,1.06 |

Table S4: Excluded Studies

| **Study number** | **Publication Year** | **First Author** | **Title** | **Exclusion Reason** |
| --- | --- | --- | --- | --- |
| 1 | 2015 | Woodhouse, Lisa | Effect of Hyperacute Administration (Within 6 Hours) of Transdermal Glyceryl Trinitrate, a Nitric Oxide Donor, on Outcome After Stroke | In-hospital research |
| 2 | 2019 | Yang, J. | Intensive ambulance-delivered blood pressure reduction in hyper-acute stroke trial | Non-RCT |
| 3 | 2018 | Wardlaw, J. M. | Effect of pre-hospital glyceryl trinitrate on hospital-based neuroimaging measures: results from the Rapid Intervention with Glyceryl trinitrate in Hypertensive stroke Trial-2 (RIGHT-2) | Non-RCT |
| 4 | 2019 | van den Berg, S. A. | Multicentre Randomised trial of Acute Stroke treatment in the Ambulance with a nitroglycerin Patch (MR ASAP): study protocol for a randomised controlled trial | Non-RCT |
| 5 | 2021 | Song, L. | INTEnsive ambulance-delivered blood pressure Reduction in hyper-ACute stroke Trial (INTERACT4): study protocol for a randomized controlled trial | Non-RCT |
| 6 | 2012 | Bath, P. M. W. | Rapid intervention with glyceryl trinitrate (GTN) in hypertensive stroke trial (RIGHT): Determining the safety of GTN and potential of ambulance-based randomised controlled trials in patients with ultra-acute stroke | Non-RCT |
| 7 | 2012 | Ankolekar, S. | Determining the Feasibility of Ambulance-Based Randomised Controlled Trials in Patients with Ultra-Acute Stroke: Study Protocol for the "Rapid Intervention with GTN in Hypertensive Stroke Trial" (RIGHT, ISRCTN66434824) | Non-RCT |
| 8 | 2012 | Ankolekar, S. | Rapid Intervention with Glyceryl trinitrate (GTN) in Hypertensive stroke Trial (RIGHT): Safety of GTN and potential of ambulance trials in ultra-acute stroke | Non-RCT |
| 9 | 2012 | Ankolekar, S. | Rapid intervention with GTN (Glyceryl Trinitrate) in hypertensive stroke trial (RIGHT): Determining the potential of ambulance-based randomised controlled trials in patients with ultra-acute stroke | Non-RCT |
| 10 | 2021 | Uniken Venema, S. | Multicentre randomised trial of acute stroke treatment in the ambulance with a nitroglycerin patch (MR ASAP) | Review |
| 11 | 2019 | Reinink, R. | Multicentre randomised trial of acute stroke treatment in the ambulance with a nitroglycerin patch (MR ASAP) | Review |
| 12 | 2022 | Tunnage, B. | Pre-hospital transdermal glyceryl trinitrate in patients with stroke mimics: data from the RIGHT-2 randomised-controlled ambulance trial | wrong patients |
| 13 | 2018 | Nut, | Intensive Ambulance-delivered Blood Pressure Reduction in Hyper-Acute Stroke Trial | wrong patients |
| 14 | 2024 | Liu, R. | Process Evaluation of an Ambulance-delivered Early Intensive Blood Pressure Lowering Stroke Trial: Design, Rationale, and Reflection | wrong patients |
| 15 | 2024 | Li, G. | Intensive Ambulance-Delivered Blood-Pressure Reduction in Hyperacute Stroke | Not retrieved |
| 16 | 2017 | Euctr, N. L. | Study on the treatment with a nitroglycerin patch in the ambulance for patients with a stroke | Not retrieved |
| 17 | 2022 | Dixon, M. | Time intervals and distances travelled for prehospital ambulance stroke care: data from the randomised-controlled ambulance-based Rapid Intervention with Glyceryl trinitrate in Hypertensive stroke Trial-2 (RIGHT-2) | Not retrieved |
| 18 | 2019 | ChiCtr, | INTEnsive ambulance-delivered blood pressure Reduction in hyper-ACute stroke Trial | Not retrieved |
| 19 | 2023 | Chen, C. | Update on the INTEnsive ambulance-delivered blood pressure Reduction in hyper-ACute stroke Trial (INTERACT4): progress and baseline features in 2053 participants | Not retrieved |
| 20 | 2023 | Chen, C. | INTEnsive ambulance-delivered blood pressure Reduction in hyper-Acute stroke Trial (INTERACT4): progress Update and Experiences during COVID | Not retrieved |
| 21 | 2023 | Chen, C. | INTENSIVE AMBULANCE-DELIVERED BLOOD PRESSURE REDUCTION IN HYPER-ACUTE STROKE TRIAL (INTERACT4): PROGRESS UPDATE AND BASELINE FEATURES OF 1387 PATIENTS | Not retrieved |
| 22 | 2024 | Billot, L. | Statistical Analysis Plan for the INTEnsive ambulance-delivered blood pressure Reduction in hyper-ACute stroke Trial (INTERACT4) | Not retrieved |
| 23 | 2018 |  | Intensive Ambulance-delivered Blood Pressure Reduction in Hyper-Acute Stroke Trial | Not retrieved |
| 24 | 2023 | Woodhouse, L. J. | Prehospital transdermal glyceryl trinitrate in patients with ultra-acute presumed stroke (RIGHT-2): Effects on outcomes at day 365 in a randomised, sham-controlled, blinded, phase III, superiority ambulance-based trial | Inconsistency |
| 25 | 2024 | Appleton, J. P. | Prehospital transdermal glyceryl trinitrate for ultra-acute ischaemic stroke: data from the RIGHT-2 randomised sham-controlled ambulance trial | Inconsistency |
| 26 | 2019 | Appleton, J. P. | Ambulance-delivered transdermal glyceryl trinitrate versus sham for ultra-acute stroke: rationale, design and protocol for the Rapid Intervention with Glyceryl trinitrate in Hypertensive stroke Trial-2 (RIGHT-2) trial (ISRCTN26986053) | Inconsistency |
| 27 | 2010 | Žižka, J. | Evaluation of cardiovascular high risk population in Specialists Ambulance: ESA | Unrelated |
| 28 | 2019 | Zhelev, Z. | Prehospital stroke scales as screening tools for early identification of stroke and transient ischemic attack | Unrelated |
| 29 | 2018 | Zhao, W. | Remote ischaemic conditioning for preventing and treating ischaemic stroke | Unrelated |
| 30 | 2021 | Zhang, Y. | Reperfusion strategy and in-hospital outcomes for ST elevation myocardial infarction in secondary and tertiary hospitals in predominantly rural central China: A multicentre, prospective and observational study | Unrelated |
| 31 | 2016 | Yperzeele, L. | Prehospital heart rate variability in patients with suspicion of acute stroke: Predictive and diagnostic value | Unrelated |
| 32 | 2009 | Yeh, H. F. | Traumatic vertebral artery dissection and Wallenberg syndrome after a motorcycle collision | Unrelated |
| 33 | 2022 | Yamaguchi, S. | Posterior reversible encephalopathy syndrome mimicking subacute ischemic stroke: a case report | Unrelated |
| 34 | 2014 | Xiang, L. | [The significance of ambulance equipped with ventilation in transportation of patients with cerebral infarction and OSAHS] | Unrelated |
| 35 | 2020 | Woodhouse, L. | Comparing neuroradiologist adjudication with automated imaging software in cerebral ischaemia, haemorrhage and mimics: from the rapid intervention with glyceryl trinitrate in hypertensive stroke trial-2 (right-2) | Unrelated |
| 36 | 2019 | Woodhouse, L. | Effect of glyceryl trinitrate on outcome after acute stroke: Update of a systematic review and metaanalysis of individual patient data | Unrelated |
| 37 | 2020 | Woodhouse, L. | Relationship between haematoma length and outcome Data from the tranexamic acid for intracerebral haemorrhage 2 (TICH-2) trial | Unrelated |
| 38 | 2016 | Weeks, G. | Non‐medical prescribing versus medical prescribing for acute and chronic disease management in primary and secondary care | Unrelated |
| 39 | 2018 | Wang, P. L. | Mechanical versus manual chest compressions for cardiac arrest | Unrelated |
| 40 | 2023 | Wan, Y. | Manifestations and Outcomes of Intracerebral Hemorrhage During the COVID-19 Pandemic in China: Multicenter, Longitudinal Cohort Study | Unrelated |
| 41 | 2010 | Walter, S. | Bringing the hospital to the patient: First treatment of stroke patients at the emergency site | Unrelated |
| 42 | 2024 | Wallis, J. A. | Factors influencing the implementation of early discharge hospital at home and admission avoidance hospital at home: a qualitative evidence synthesis | Unrelated |
| 43 | 2024 | Voigt, S. | Stopping haematoma growth: the search for the right time, place, and agent | Unrelated |
| 44 | 2020 | Tunnage, B. | Pre-hospital transdermal glyceryl trinitrate in patients with stroke mimics: A sub-group analysis of the rapid intervention with glyceryl trinitrate in hypertensive stroke trial-2 (Right-2) | Unrelated |
| 45 | 2013 | Tsukinoki, R. | Non-communicable disease epidemic: epidemiology in action (EuroEpi 2013 and NordicEpi 2013): Aarhus, Denmark from 11 August to 14 August 2013 | Unrelated |
| 46 | 2018 | Suzuki, J. | Ten-Year Evaluation of the TOYOTA Prehospital Stroke Scale for Tissue Plasminogen Activator Intravenous Therapy in the Real World | Unrelated |
| 47 | 2013 | Sun, X. G. | Public and professional education on urgent therapy for acute ischemic stroke: A community-based intervention in Changsha | Unrelated |
| 48 | 2024 | Stuby, L. | A Two-Step Approach Using the National Health Institutes of Health Stroke Scale Assessed by Paramedics to Enhance Prehospital Stroke Detection: A Case Report and Concept Proposal | Unrelated |
| 49 | 2018 | Strøm, C. | Hospitalisation in short‐stay units for adults with internal medicine diseases and conditions | Unrelated |
| 50 | 2015 | Sprigg, N. | Glyceryl trinitrate for hyperacute stroke: Results from the efficacy of nitric oxide in stroke (ENOS) trial | Unrelated |
| 51 | 2019 | Song, L. | Novel clinical trial design: interact4, an ambulance-delivered probe trial of intensive bp lowering | Unrelated |
| 52 | 2022 | Song, L. | INTENSIVE AMBULANCE-DELIVERED BLOOD PRESSURE REDUCTION IN HYPER-ACUTE STROKE TRIAL (INTERACT4): PROGRESS UPDATE | Unrelated |
| 53 | 2019 | Song, L. | Intensive ambulance-delivered blood pressure reduction in hyper-acute stroke trial (INTERACT4) | Unrelated |
| 54 | 2012 | Sheikh, A. | Adrenaline auto‐injectors for the treatment of anaphylaxis with and without cardiovascular collapse in the community | Unrelated |
| 55 | 2014 | Shaw, L. | Paramedic Initiated Lisinopril for Acute Stroke Treatment (PIL-FAST): Results from the pilot randomised controlled trial | Unrelated |
| 56 | 2011 | Shaw, L. | Paramedic Initiated Lisinopril For Acute Stroke Treatment (PIL-FAST): Study protocol for a pilot randomised controlled trial | Unrelated |
| 57 | 2018 | Scutt, P. | Statistical analysis plan for the ‘Rapid Intervention with Glyceryl trinitrate in Hypertensive stroke Trial-2 (RIGHT-2)’ | Unrelated |
| 58 | 2024 | Schwabauer, E. | Effects of Mobile Stroke Unit dispatch on blood pressure management and outcomes in patients with intracerebral haematoma: Results from the Berlin_Prehospital Or Usual Care Delivery in acute Stroke (B_PROUD) controlled intervention study | Unrelated |
| 59 | 2012 | Schmidt, M. R. | Remote ischemic conditioning: The cardiologist's perspective | Unrelated |
| 60 | 1997 | Schaarschmidt, S. | Acute theophylline intoxication as a differential diagnosis to a pneumothorax in an asthmatic patient | Unrelated |
| 61 | 2010 | Sanossian, N. | Development and validation of a clinical scale distinguishing hemorrhagic and ischemic stroke in the first two hours after onset | Unrelated |
| 62 | 2013 | Sanossian, N. | Enrolling spanish-speaking subjects into prehospital research: The FAST-MAG spanish line experience | Unrelated |
| 63 | 2012 | Sanossian, N. | Field neuroprotective therapy followed by intravenous thrombolysis in a phase 3 clinical trial | Unrelated |
| 64 | 2015 | Rinehart, D. R. | Management of Apixaban-Associated Subdural Hematoma: A Case Report on the Use of Factor Eight Inhibitor Bypassing Activity | Unrelated |
| 65 | 2023 | Riera-López, N. | Effect of the COVID-19 pandemic on advanced life support units’ prehospital management of the stroke code in four Spanish regions: an observational study | Unrelated |
| 66 | 2024 | Putrik, P. | Models for delivery and co‐ordination of primary or secondary health care (or both) to older adults living in aged care facilities | Unrelated |
| 67 | 2020 | Purroy, F. | REMOTE Ischemic Perconditioning Among Acute Ischemic Stroke Patients in Catalonia: REMOTE-CAT PROJECT | Unrelated |
| 68 | 2017 | Pantoja, T. | Implementation strategies for health systems in low‐income countries: an overview of systematic reviews | Unrelated |
| 69 | 2020 | Odendaal, W. A. | Health workers’ perceptions and experiences of using mHealth technologies to deliver primary healthcare services: a qualitative evidence synthesis | Unrelated |
| 70 | 2023 | Nct, | Cardiac Arrest Bundle of cARE Trial | Unrelated |
| 71 | 2013 | Nct, | Community Level Interventions for Pre-eclampsia | Unrelated |
| 72 | 2013 | Nct, | Passive Leg Raise (PLR) During Cardiopulmonary Resuscitation (CPR) | Unrelated |
| 73 | 2010 | Nct, | Paramedic Initiated Lisinopril For Acute Stroke Treatment | Unrelated |
| 74 | 2007 | Nct, | Remote Ischemic Preconditioning in Primary PCI | Unrelated |
| 75 | 2002 | Nct, | Progesterone Treatment of Blunt Traumatic Brain Injury | Unrelated |
| 76 | 2020 | Nan, J. | Comparison of clinical outcomes in patients with ST elevation myocardial infarction with percutaneous coronary intervention and the use of a telemedicine app before and after the COVID-19 pandemic at a Center in Beijing, China, from August 2019 to March 2020 | Unrelated |
| 77 | 2022 | Montgomery, G. W. A. | Retching as an unusual cause of bilateral posterior shoulder fracture dislocation | Unrelated |
| 78 | 2013 | McMullan, D. M. | Expanding the availability of extracorporeal cardiopulmonary resuscitation | Unrelated |
| 79 | 2023 | Mack, D. | Extraocular sebaceous carcinoma of the chest wall: A case report | Unrelated |
| 80 | 2023 | Lynch, E. A. | Interventions for the uptake of evidence‐based recommendations in acute stroke settings | Unrelated |
| 81 | 2022 | Lindvåg Lie, S. | Effects of supplemental oxygen on systemic and cerebral hemodynamics in experimental hypovolemia: protocol for a randomized, double blinded crossover study | Unrelated |
| 82 | 2014 | Lim, W. | Stroke literacy in Singapore: data from a survey of public housing estate residents | Unrelated |
| 83 | 2021 | Li, Z. | Application analysis of 5G mobile stroke unit in stroke screening and emergency treatment | Unrelated |
| 84 | 2023 | Larsen, K. T. | Antithrombotic Treatment, Prehospital Blood Pressure, and Outcomes in Spontaneous Intracerebral Hemorrhage | Unrelated |
| 85 | 2018 | Kobayashi, A. | European Academy of Neurology and European Stroke Organization consensus statement and practical guidance for pre-hospital management of stroke | Unrelated |
| 86 | 2015 | Khor, M. X. | Pre-hospital notification is associated with improved stroke thrombolysis timing | Unrelated |
| 87 | 2017 | Kettner, M. | Prehospital Computed Tomography Angiography in Acute Stroke Management | Unrelated |
| 88 | 2023 | Kapoor, S. | CRANIAL ULTRASOUND FOR PREHOSPITAL INTRACEREBRAL HEMORRHAGE DETECTION: EXPLORATORY FEASIBILITY STUDY | Unrelated |
| 89 | 2018 | Isrctn, | Acute stroke treatment in the ambulance with a nitroglycerin patch | Unrelated |
| 90 | 2017 | Isrctn, | Is intravenous alteplase still of added benefit in patients with acute ischaemic stroke who undergo intra-arterial treatment? | Unrelated |
| 91 | 2017 | Ishihara, H. | Safety and Time Course of Drip-and-Ship in Treatment of Acute Ischemic Stroke | Unrelated |
| 92 | 2010 | Hougaard, K. D. | Remote ischemic perconditionering in acute stroke: An endogeneous model to generate neuroprotection | Unrelated |
| 93 | 2019 | Hansson, P. O. | Prehospital assessment of suspected stroke and TIA: An observational study | Unrelated |
| 94 | 2017 | Glezer | Adherence of Patients to Antihypertensive Therapy With Combination of Perindopril and Indapamide According to Data of the FORSAGE Program | Unrelated |
| 95 | 2012 | Gillespie, L. D. | Interventions for preventing falls in older people living in the community | Unrelated |
| 96 | 2018 | Fritzen, K. | Improvement of Metabolic Control and Diabetes Management in Insulin-Treated Patients Results in Substantial Cost Savings for the German Health System | Unrelated |
| 97 | 2017 | Fassbender, K. | Mobile stroke units for prehospital thrombolysis, triage, and beyond: benefits and challenges | Unrelated |
| 98 | 2008 | Euctr, G. B. | Rapid Intervention with GTN in Hypertensive stroke Trial (RIGHT). Determining the potential of ambulance-based randomised controlled trials in patients with hyperacute stroke; assessment of glyceryl trinitrate in lowering blood pressure. - RIGHT | Unrelated |
| 99 | 2024 | Endo, H. | Cerebral hyperperfusion syndrome after endovascular reperfusion therapy for medium vessel occlusion: A case report | Unrelated |
| 100 | 2024 | Edlow, J. A. | Lowering Blood Pressure in Stroke Patients in the Ambulance - A Bridge Too Close? | Unrelated |
| 101 | 2015 | Ebinger, M. | Mobile computed tomography: Prehospital diagnosis and treatment of stroke | Unrelated |
| 102 | 2024 | Dixon, M. | Challenges and Experiences in Multicenter Prehospital Stroke Research: Narrative Data from the Rapid Intervention with Glyceryl Trinitrate in Hypertensive Stroke Trial-2 (RIGHT-2) | Unrelated |
| 103 | 2005 | de Keyser, J. | Neuroprotection in acute ischemic stroke | Unrelated |
| 104 | 2013 | Capoulade, R. | Low stroke volume index provides incremental prognostic value beyond left ventricular ejection fraction in patients with aortic stenosis | Unrelated |
| 105 | 2018 | Cameron, I. D. | Interventions for preventing falls in older people in care facilities and hospitals | Unrelated |
| 106 | 2016 | Cabello, J. B. | Oxygen therapy for acute myocardial infarction | Unrelated |
| 107 | 1997 | Burroughs, A. K. | Optimising emergency care of upper gastrointestinal bleeding in cirrhotic patients | Unrelated |
| 108 | 2022 | Bugge, H. F. | National Institutes of Health Stroke Scale scores obtained using a mobile application compared to the conventional paper form: a randomised controlled validation study | Unrelated |
| 109 | 2002 | Brown, M. M. | Brain attack: a new approach to stroke | Unrelated |
| 110 | 2023 | Blauenfeldt, R. A. | Remote Ischemic Conditioning for Acute Stroke: the RESIST Randomized Clinical Trial | Unrelated |
| 111 | 2019 | Bath, P. M. | The Rapid Intervention with Glyceryl trinitrate in Hypertensive stroke Trial-2 (RIGHT-2): Results from the 1 year follow-up | Unrelated |
| 112 | 2019 | Bath, P. M. | Prehospital Transdermal Glyceryl Trinitrate for Ultra-Acute Intracerebral Hemorrhage: data From the RIGHT-2 Trial | Unrelated |
| 113 | 2019 | Bath, P. M. | Glyceryl trinitrate (GTN) for prehospital ultra-acute intracerebral haemorrhage (ICH): A sub-group analysis of the rapid intervention with glyceryl trinitrate in hypertensive stroke trial-2 (right-2) | Unrelated |
| 114 | 2018 | Bath, P. M. | Glyceryl trinitrate for pre-hospital ultra-acute stroke: Main results from the Rapid Intervention with Glyceryl trinitrate in Hypertensive stroke Trial-2 (RIGHT-2) | Unrelated |
| 115 | 2019 | Bath, P. M. | Baseline characteristics of the 1149 patients recruited into the Rapid Intervention with Glyceryl trinitrate in Hypertensive stroke Trial-2 (RIGHT-2) randomized controlled trial | Unrelated |
| 116 | 2015 | Bath, P. | Rapid intervention with glyceryl trinitrate in hypertensive stroke trial-2 (RIGHT-2) | Unrelated |
| 117 | 2016 | Bath, P. | Rapid intervention with glyceryl trinitrate in hypertensive stroke trial-2 (right-2): Safety and efficacy of transdermal glyceryl trinitrate, a nitric oxide donor | Unrelated |
| 118 | 2018 | Bath, P. | Rapid intervention with glyceryl trinitrate in hypertensive stroke trial-2 (right-2): Safety and efficacy of transdermal glyceryl trinitrate, a nitric oxide donor | Unrelated |
| 119 | 2018 | Bath, P. | Rapid intervention with glyceryl trinitrate in hypertensive stroke trial-2 (right-2): safetyand efficacy of transdermal glyceryl trinitrate, a nitric oxide donor | Unrelated |
| 120 | 2023 | Alshehri, A. | PHYSIOLOGICAL VARIABILITY DURING PREHOSPITAL STROKE CARE - WHAT MONITORING AND INTERVENTIONS ARE USED? | Unrelated |
| 121 | 2022 | van den Berg, S. A. | Prehospital transdermal glyceryl trinitrate in patients with presumed acute stroke (MR ASAP): an ambulance-based, multicentre, randomised, open-label, blinded endpoint, phase 3 trial. | include |
| 122 | 2024 | Li, G. | Intensive Ambulance-Delivered Blood-Pressure Reduction in Hyperacute Stroke. | include |
| 123 | 2013 | Ankolekar, S. | Feasibility of an ambulance-based stroke trial, and safety of glyceryl trinitrate in ultra-acute stroke: the rapid intervention with glyceryl trinitrate in Hypertensive Stroke Trial (RIGHT, ISRCTN66434824). | include |
| 124 | 2019 | Bath, PM | Prehospital transdermal glyceryl trinitrate in patients with ultra-acute presumed stroke (RIGHT-2): an ambulance-based, randomised, sham-controlled, blinded, phase 3 trial. | include |

Figure S1: Risk of bias summary.


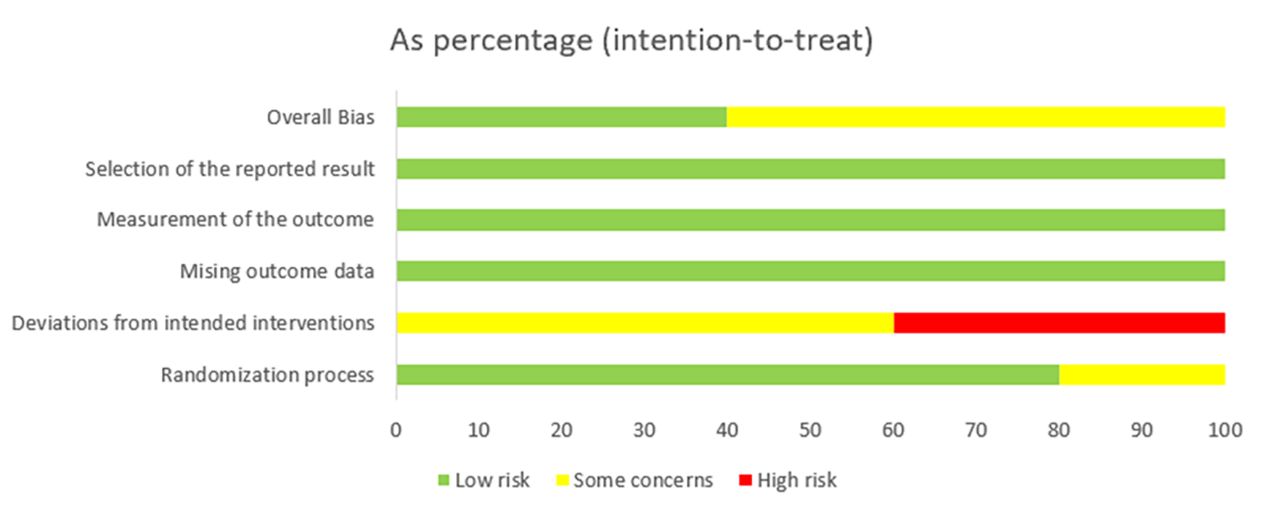


Figure S2: Risk of bias graph.


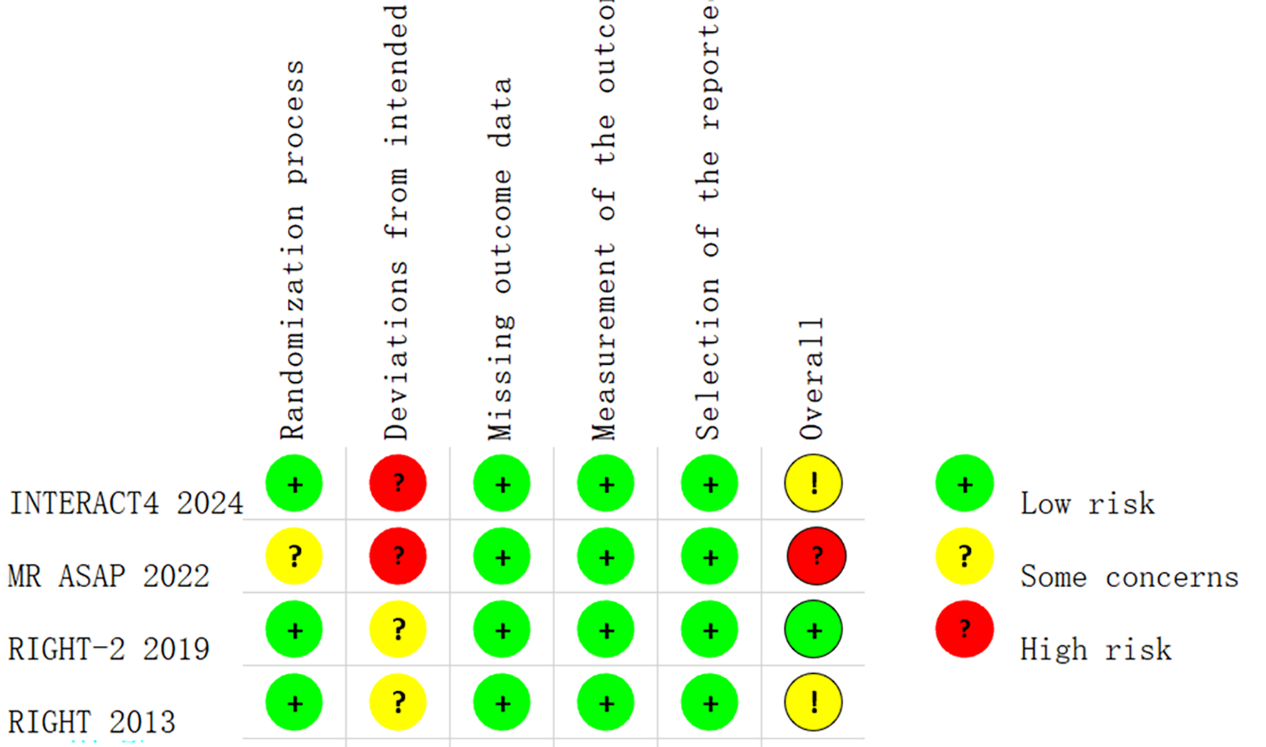


Figure S3: Subgroup analysis of the association between ambulance-intensive blood pressure lowering therapy and poor prognosis.


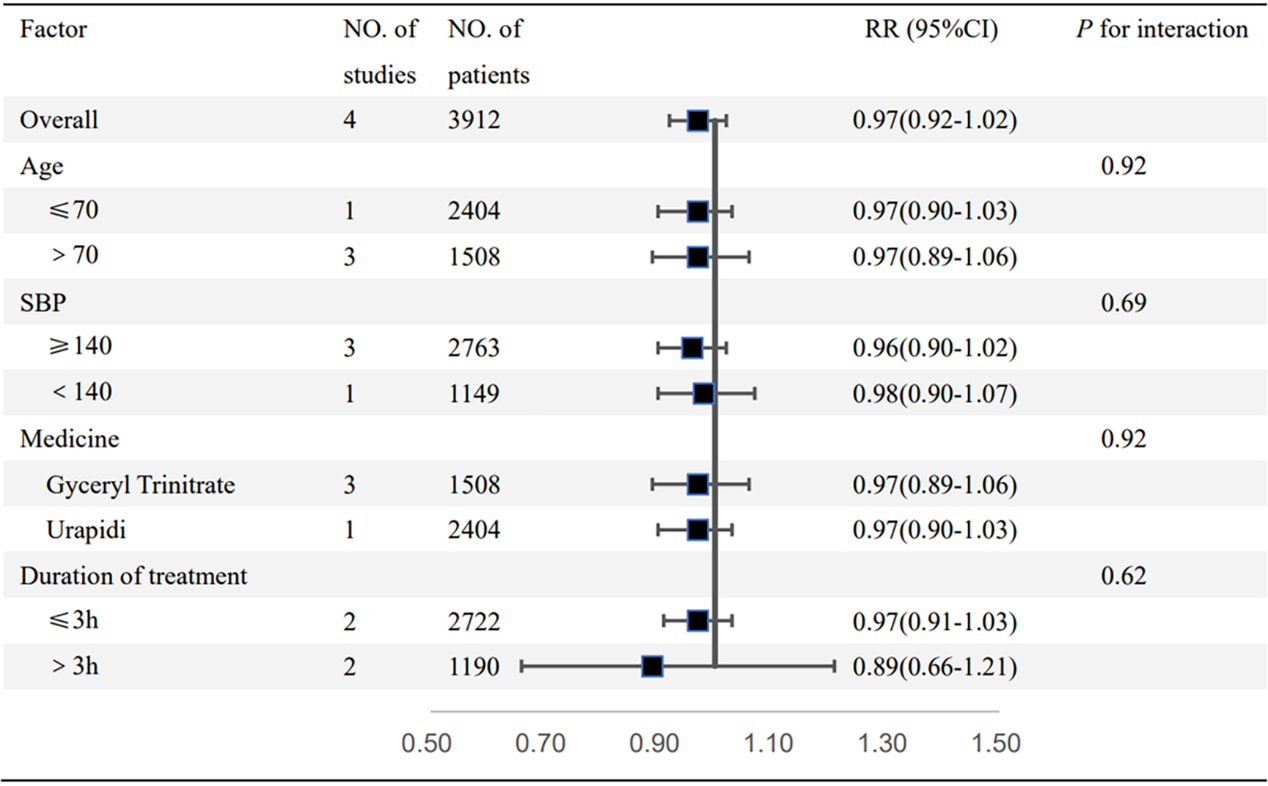


Cl: confidence interval, *P*: *P* for interaction for subgroup difference; SBP: Systolic Blood Pressure.
